# Post-acute sequelae of SARS-CoV-2 infection (PASC): peripheral, autonomic, and central nervous system features in a child

**DOI:** 10.1007/s10072-021-05345-5

**Published:** 2021-07-11

**Authors:** David S. Younger

**Affiliations:** Department of Medicine, Division of Neuroscience, City University of New York Medical School, 333 East 34th Street, 1J, New York, NY 10016 USA

## Introduction

The 2019 novel coronavirus (2019‐nCoV [COVID-19]) epidemic is caused by the novel severe acute respiratory syndrome-coronavirus-2 (SARS-nCoV-2 or SARS-CoV-2). There are reviews of the acute neurological illness in adults [1] and children [2]. Recent attention has also been focused on the post-acute sequelae of SARS-CoV-2 infection (PASC) by the National Institutes of Health (NIH) through its launch of the Research Opportunity Announcement (ROA) OTA-21–0158, and the formation of the SARS-CoV-2 Recovery Cohort and Investigator Consortium (https://covid19.nih.gov/sites/default/files/2021-02/PASC-ROA-OTA-Recovery-Cohort-Studies.pdf). The NIH operationally defines PASC as the failure to recover from acute COVID-19, or those persistently symptomatic for > 30 days from onset of infection, with any pattern of tissue injury that remains evolving including the nervous system. Online hosted surveys enrolling thousands of subjects employing mobile apps for symptom tracking to obtain real-world data and evidence provide useful information substantiating PASC in adults [3] but exclude subjects < 18 years old. While awaiting prospective NIH-funded research, there is an urgency to understand PASC in children.

This paper describes the detailed evaluation and treatment of an adolescent with PASC manifesting progressive post-infectious central, peripheral, and autonomic nervous system (CNS, PNS, and ANS).

## Materials and methods

The initial diagnosis of acute COVID-19 infection and immunity was confirmed by a positive reverse-transcriptase polymerase-chain-reaction (RT-PCR) on a nasopharyngeal swab, and acquired immunity was ascertained by the presence of elevated serum SARS-CoV-2 IgG-specific antibody. Electrodiagnostic studies (EDX) were performed in three limbs according to standard methods [4]. Epidermal nerve fiber (ENF) densities were ascertained in a 3-mm punch biopsy of skin taken from the lateral thigh and calf and placed in paraformaldehyde-lysine-periodic acid fixative and compared to age-matched controls using normative data [5, 6]. Autonomic evaluation included beat-to-beat blood pressure acquisition for testing of cardiovagal (parasympathetic) function, adrenergic (sympathetic) function of heart rate (HR) and systolic blood pressure (SBP) during rapid respiration, Valsalva maneuver and 5-min 70° head up tilting (HUT) using autonomic laboratory components (WR Medical Electronics, MN) were performed according to standard guidelines [7]. ^18^Fluorodeoxyglucose (FDG) positron emission tomography (PET) fused to 3-Tesla non-contrast brain magnetic resonance imaging (MRI) with 3D rendering was performed using volumetric software. Mayo Clinic (Mayo Labs, Rochester, MN) serum autoantibody (ENS2) panel screened for autoimmune encephalopathy. Twenty-channel electroencephalography (EEG) was performed in the awake and drowsy states. Lumbar cerebrospinal fluid (CSF) was assayed for infectious, immunological, and cytologic parameters.

## Results

**Fig. 1:**
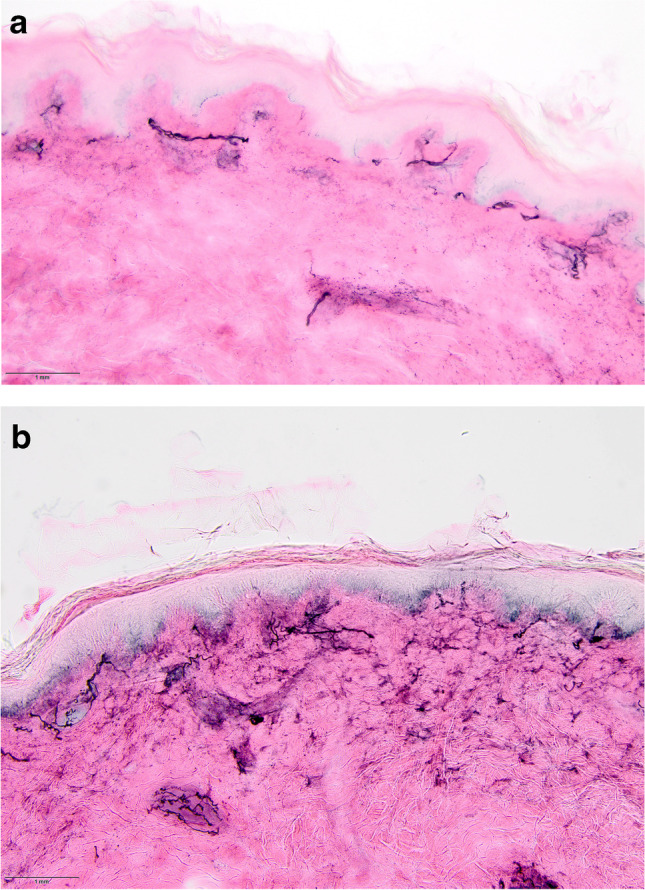
Immunohistochemical staining for ENF using the PGP9.5-antibody, counter-stained by Eosin from the skin of the left calf and thigh. **a** Photomicrograph of left calf ENFs with a density of 8.9 ENFs. **b** Photomicrograph of left thigh ENFs with a density of 8.9 ENFs/mm skin. Original magnifications × 200. Courtesy of Kurenai Tanji MD, Department of Pathology and Cell Biology, Columbia University, New York. Abbreviations: ENF, epidermal nerve fiber; PGP9.5, protein gene product 9.5

**Fig. 2:**
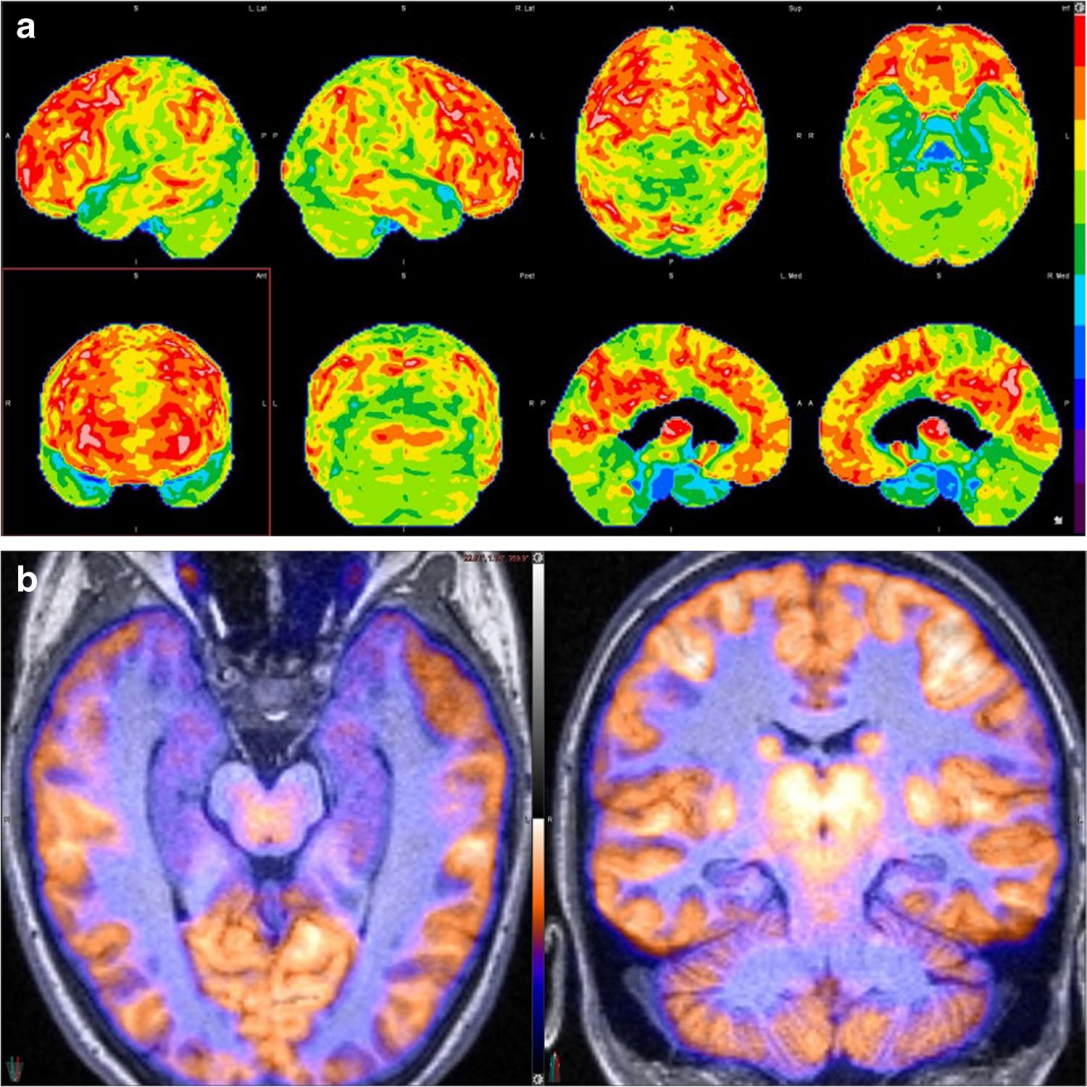
**a** 3D-SSP analysis of ^18^FDG PET normalized to the whole brain shows mild-to-moderate FDG hypometabolism in the bilateral anterior and medial temporal lobes, superior parietal lobes, lateral occipital lobes, anterior cingulate cortices, and bilateral cerebellar hemispheres. **b** ^18^FDG PET/MRI fused image clearly shows the degree of FDG hypometabolism and volume loss in the bilateral hippocampi. Courtesy of Elcin Zan MD, Department of Radiology (Neuroradiology), New York University Grossman School of Medicine, New York. Abbreviations: 3D-SSP, three-dimensional stereotactic surface projection; ^18^FDG, ^18^Fluorodeoxyglucose; PET, positron emission tomography; MRI, magnetic resonance imaging

A 12-year-old girl without significant past medical history was initially well until March 2020 when following exposure to other family members who tested positive for COVID-19 infection she contracted an upper respiratory infection (URI), loss of taste, and excessive fatigue. She failed to recover by July 2020 while developing progressive burning pain, limb weakness, and slurred speech and impaired cognition, such that by July 2020 she was bedbound. There was no history of seizures. There was concern that she might be suffering from a conversion disorder. Examination in September 2020 showed mild delirium. There was weakness versus gravity in the legs, resistance in the arms, with stocking sensory loss to vibration, cold temperature, and pin prick, and areflexia. EDX showed mixed chronic distal demyelinating and axonal changes. Thigh ENF density was 16.3 ENF/mm skin (normal 31.6 ± 13.2) and calf ENF density was 8.9 ENF/mm (normal 20.3 ± 7.4) (Fig. 1). HUT showed resting tachycardia of 118 bpm, with severe symptomatic orthostatic intolerance associated with a sustained fall in SBP to 63.25 mmHg (delta of 25.75 mmHg) so noted at 0.24 min following HUT, and further HR acceleration to 145 bpm (delta of change of 25 bpm) with HUT. PET/MRI showed hypometabolism of bilateral anterior and mesial temporal, superior parietal, and lateral occipital lobes, anterior cingulate cortices, and the cerebellar hemispheres (Fig. 2). Volumetric analysis suggested left hippocampal and temporal hippocampal volumes to be < 5% of age-matched controls. Mayo Clinic ENS2 panel showed a serum glutamic acid dehydrogenase (GAD)65 antibody titer of 0.03 nmol/L (reference value ≤ 0.02). Lumbar puncture showed a total protein of 136 mg/dL, otherwise normal. EEG showed no evidence of localized slowing or epileptiform activity. From October 2020 to February 2021, she received 2 g/kg/month of intravenous immune globulin (IVIg) therapy with overall initial clinical improvement.

## Discussion

Progressive PNS, ANS, and CNS involvement developed in a child recovering from COVID-19 presenting with a URI. There are several reasons to consider an autoimmune etiopathogenesis related to preceding SARS-CoV-2 infection. First, children with or without multisystem inflammatory syndrome (MIS-C), as in this patient, exhibit similar pathogenic SARS-CoV-2 IgG anti-spike (S) antibody profiles with comparatively lower levels of neutralizing activity and anti-nucleocapsid (N)-specific antibodies associated with a reduced protective serologic response [8]. Second, absence of the acute respiratory distress syndrome (ARDS) in this patient and in children overall is consistent with the lower expression of SARS-CoV-2 viral receptor (angiotensin-converting enzyme 2 (ACE2)) in airway epithelial cells [9], yet a more robust innate immune response [10]. Third, pediatric T-cell responses to SARS-CoV-2 exceed the adult responses due to an increased number of naïve T-cells available to respond to new pathogens [11] including those recently acquired T-cell memory cells related to coronavirus strains [12] and more frequent respiratory illnesses seen in children overall. Fourth, there is mounting interest in the contribution of infection and the host microbiome [13] and antineuronal antibodies to neuropsychiatric illness [14] particularly autoantibodies reactive to surface antigens of neurons in the limbic system present in the hippocampus, that are important in mood and cognition.

### Central nervous system involvement

There are no reported studies of brain PET/MRI studies in pediatric COVID-19 neurological illness; however, affected adults show hypometabolism in multiple cortical areas in acute [15] and late COVID-19 illness [16], likely reflecting impaired neuronal activity [17]. Putative mechanisms of COVID-19 CNS involvement including encephalopathy include a dysregulated neuroinflammatory immune response to the systemic infection as well as the impact of pre-existing medical conditions, rather than virally mediated pathology despite detection of SARS-CoV-2 RNA and proteins in postmortem brain tissues [18]. Although there are no postmortem series of childhood COVID-neurological illness, adult COVID-19 decedents [19] show elevated levels of circulating interleukin (IL)-6, IL-8, and tumor necrosis factor (TNF)-α, indicative of a cytokine storm associated with focal and diffuse cortical, brainstem, and leptomeningeal T-cell mediated inflammation.

### Peripheral nervous system involvement

LaRovere and colleagues [20] described 4 hospitalized children with COVID-19 manifesting new deficits of Guillain-Barré syndrome (GBS) at discharge; 2 others with painful neuropathy and neurocognitive deficits (2 cases) reminiscent of the present patient. Dalakas [21] suggested a post-infectious autoimmune mechanism in 11 cases of GBS lacking evidence of SARS-CoV-2 replication in CSF, and associated with GD1b antibodies in another, all with improvement after IVIg. GBS in association with COVID-19 was heralded by fever (74%), cough (67%), interstitial pneumonia (62%), hypoageusia (38%), and hypoanosmia (26%) in a meta-analysis of 42 adults [22] and in rare instances of childhood affliction [23] that would qualify for PASC.

### Autonomic involvement

Dysautonomia has been reported in COVID-19-related GBS [24] with frequent association of sphincter dysfunction. The present patient instead manifested dysautonomia as a feature of painful small fiber sensory neuropathy (SFN) [25] shown respectively by tilt table testing and ENF histology. Similarly affected adults recovering from COVID-19 were successfully treated with IVIg [26]. New-onset postural orthostatic tachycardia syndrome (POTS) was described in a previously healthy adult patient weeks after quarantining from COVID-19 illness suggesting an immune mechanism of onset [27] but not in pediatric subjects. SARS-CoV-2 tropism for the brainstem due to increased expression of ACE2 and corresponding neuroinflammatory changes associated with COVID-19 neurological illness [28] may contribute to persistent brainstem dysfunction and latent dysautonomia. There is a potential role for cardiac imaging with ^123^I-meta-iodobenzylguanidine (MIBG) in suspected cases of sympathetic denervation associated with dysautonomia in PASC to assess the integrity of the cardiac ANS [29].

### Toward a unified concept of PASC

PASC can be described by the acronym, I-Cubed (I^3^) that posits a multiplier effect of infection, immunity, and inflammation; which conditioned by environmental and genetic predisposing factors, may be the source of host autoimmunity [30]. The underlying basis of PASC, especially in the CNS, may not be fully appreciated until controlled clinical and autopsy cohort studies are performed. IVIg appears to be an effective agent in the management of MIS-C and Kawasaki disease (KD) which it can mimic [31] as well as adult PASC [16]. It is unlikely that the designations COVID Long Hauler and Long COVID will be appropriate terms for PASC. However, given the need to assemble 20,000 subjects with SARS-CoV-2 infection and 1000 subjects with PASC for the NIH-funded initiatives, their sheer magnitude and reach of social media may make them useful cohorts for future controlled research.
